# Loss of adipose triglyceride lipase is associated with human cancer and induces mouse pulmonary neoplasia

**DOI:** 10.18632/oncotarget.9418

**Published:** 2016-05-17

**Authors:** Wael Al-Zoughbi, Martin Pichler, Gregor Gorkiewicz, Barbara Guertl-Lackner, Johannes Haybaeck, Stephan W. Jahn, Carolin Lackner, Bernadette Liegl-Atzwanger, Helmut Popper, Silvia Schauer, Elisa Nusshold, Alida S. D. Kindt, Zlatko Trajanoski, Michael R. Speicher, Guenther Haemmerle, Robert Zimmermann, Rudolf Zechner, Paul W. Vesely, Gerald Hoefler

**Affiliations:** ^1^ Institute of Pathology, Medical University of Graz, Graz, Austria; ^2^ Division of Oncology, Medical University of Graz, Graz, Austria; ^3^ Biocenter, Division of Bioinformatics, Medical University of Innsbruck, Innsbruck, Austria; ^4^ Institute of Human Genetics, Medical University of Graz, Graz, Austria; ^5^ Institute of Molecular Biosciences, University of Graz, Graz, Austria

**Keywords:** adipose triglyceride lipase, cancer metabolism, lipolysis, diagnostic marker

## Abstract

Metabolic reprogramming is a hallmark of cancer. Understanding cancer metabolism is instrumental to devise innovative therapeutic approaches. Anabolic metabolism, including the induction of lipogenic enzymes, is a key feature of proliferating cells. Here, we report a novel tumor suppressive function for adipose triglyceride lipase (ATGL), the rate limiting enzyme in the triglyceride hydrolysis cascade.

In immunohistochemical analysis, non-small cell lung cancers, pancreatic adenocarcinoma as well as leiomyosarcoma showed significantly reduced levels of ATGL protein compared to corresponding normal tissues. The *ATGL* gene was frequently deleted in various forms of cancers. Low levels of *ATGL* mRNA correlated with significantly reduced survival in patients with ovarian, breast, gastric and non-small cell lung cancers. Remarkably, pulmonary neoplasia including invasive adenocarcinoma developed spontaneously in mice lacking ATGL pointing to an important role for this lipase in controlling tumor development.

Loss of ATGL, as detected in several forms of human cancer, induces spontaneous development of pulmonary neoplasia in a mouse model. Our results, therefore, suggest a novel tumor suppressor function for ATGL and contribute to the understanding of cancer metabolism. We propose to evaluate loss of ATGL protein expression for the diagnosis of malignant tumors. Finally, modulation of the lipolytic pathway may represent a novel therapeutic approach in the treatment of human cancer.

## INTRODUCTION

Altered metabolism is regarded a hallmark in the malignant transformation of cells [1–3]. Specific adaptations in anabolic pathways supply rapidly proliferating cells with building blocks needed to produce nucleic acids, proteins and lipids, driving the formation of biomass [2, 4, 5]. The “classical” example for a metabolic switch in carcinogenesis is the Warburg effect [6, 7]. Other metabolic switches in the course of malignant transformation include alterations of citrate cycle enzyme activities, anabolic amino acid pathways, nucleic acids synthesis and lipid metabolism [1, 2, 4, 5, 8]. Since proliferating cancer cells require high amounts of lipids to build up biomembranes, they exhibit high rates of *de novo* lipogenesis accompanied by increased expression of lipogenic enzymes such as fatty acid synthase [9–13]. Therefore, targeting “lipid addiction” of cancer cells by inhibiting *de novo* lipid synthesis, represents a novel anti-cancer strategy [14–16]. However, detailed information on the catabolic arm of lipid metabolism during malignant transformation is lacking; insights into a possible role of lipolytic enzymes in cancer development may lead to the development of innovative therapeutic applications.

In differentiated cells, potential alternative sources of fatty acids (FA) include the hydrolytic cleavage (lipolysis) of triglycerides (TG) stored in cytoplasmic lipid droplets [12]. Although adipose tissue is the most efficient organ to store fat, essentially all other cell types can also deposit FA as TG. Upon demand, TG are hydrolyzed to provide FA for energy production, lipid synthesis, and many other cellular processes [17]. The principal enzymes for the hydrolysis of cellular TG (lipolysis) are adipose triglyceride lipase (ATGL), hormone-sensitive lipase (HSL), and monoglyceride lipase (MGL). In a consecutive manner, they hydrolyze TG, diacylglycerol and monoacylglycerol to yield FA and glycerol [18].

Nomura and colleagues recently highlighted the importance of lipases in cancer by describing the function of MGL in a FA network that promotes cancer pathogenesis [19]. In another study, the deficiency of ATGL coactivator, α/β-hydrolase domain-containing 5 (ABHD5/CGI58), resulted in increased tumorigenesis and malignant transformation [20]. Yet, the role of ATGL - the first and rate limiting enzyme in TG hydrolysis [21] - has been investigated only in cancer cell lines thus far [22]. In this study we explored whether ATGL is altered in human cancers and investigated the consequence of constitutive ATGL loss in a mouse model.

## RESULTS

The tightly regulated balance between anabolic and catabolic pathways in TG/FA cycling is crucial for cellular homeostasis [17, 18], and the expression pattern of the enzymes involved varies depending on the state of cellular differentiation. Inspection of the human proteome map portal revealed that key lipogenic enzymes are more abundant than key lipolytic enzymes in fetal organs. Remarkably, ATGL protein was undetectable in any of the fetal organs (Supplementary Figure 1, A) [23]. When we examined ATGL protein distribution in normal human adult tissues using immunohistochemical analysis, we observed an inverse correlation between ATGL protein levels and tissue proliferative capacity (Supplementary Figure 1, B). In contrast to the levels in labile cells, such as colon and skin epithelia, ATGL protein was abundant in quiescent cells, such as bronchial epithelium and smooth muscle cells, as well as in permanent cells (e.g. myocytes).

**Figure 1:**
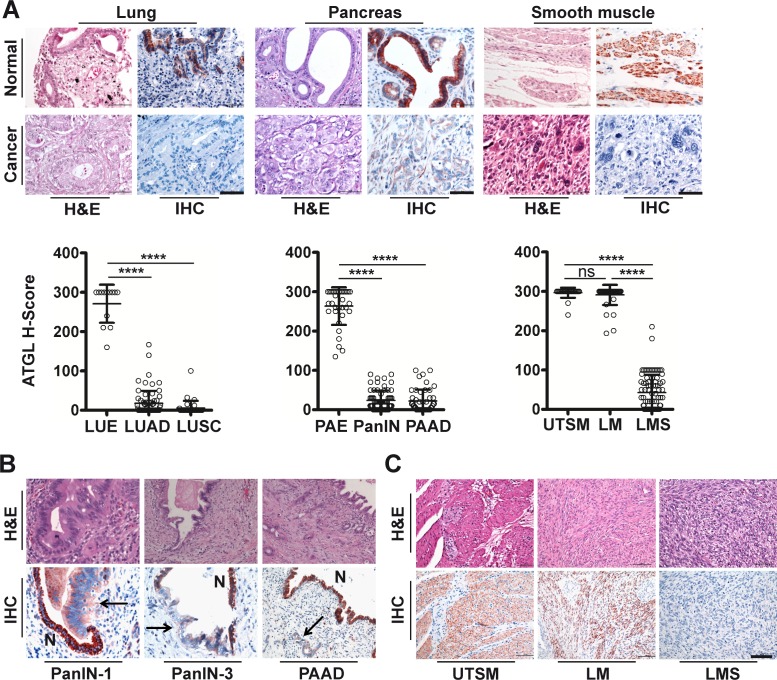
ATGL protein is frequently reduced in human cancer **A.** Representative images show haematoxylin and eosin (H&E) staining and immunohistochemical analysis (IHC) using antibodies against ATGL. Normal tissues (upper panels) are compared to cancers (lower panels). H-Score of ATGL protein levels (diagrams) determined by IHC takes into account the proportion (P, 0-100) of positive cells and the respective intensity score (I, 0-3) according to the formula [(I(0)xP(0))+(I(1)xP(1))+(I(2)xP(2))+(I(3)xP(3))]. LUE, normal lung epithelium; LUAD, lung adenocarcinoma; LUSC, lung squamous cell carcinoma; PAE, normal pancreatic epithelium; PanIN, pancreatic intraepithelial neoplasia; PAAD, pancreatic adenocarcinoma; UTSM, normal uterus smooth muscle; LM, benign tumor of smooth muscle (leiomyoma); LMS, malignant tumor of smooth muscle (leiomyosarcoma). ****P < .0001. Error bars represent SD. (LUE: *mean* = 13.47; *n* =13. LUAD: *mean* = 17.31; *n* = 70. LUSC: *mean* = 5.623; *n* = 34. PAE: *mean* = 263.4; *n* = 34. PAAD: *mean* = 22.02; *n* = 47. PanIN: *mean* = 23.48; *n* = 66. UTSM: *mean* = 296.5; *n* =26. LMS: *mean* = 42.53; *n* = 83. LM: *mean* = 291.0; *n* = 42). Scale bar, 50μm. **B.** Representative images show H&E staining and IHC using antibodies against ATGL of preinvasive neoplastic precursor lesions and invasive adenocarcinoma of the pancreas. PanIN-1, pancreatic intraepithelial neoplasia (low grade); PanIN-3, pancreatic intraepithelial neoplasia (high grade); PAAD, pancreatic adenocarcinoma. Arrows point to neoplastic lesions and invasive adenocarcinoma; N, normal epithelium. Normal epithelium stain positive for ATGL. **C.** Representative images show H&E and IHC using antibodies against ATGL. Normal smooth muscle and leiomyoma cells stain positive for ATGL whereas leiomyosarcoma cells are negative. Scale bar, 50μm.

Next, we assessed whether ATGL expression is altered in human malignancy, using ATGL immunohistochemical analysis on epithelial and mesenchymal tumors. We consistently observed low to undetectable ATGL signals in adenocarcinoma and squamous cell carcinoma of the lung that was in clear contrast to strong staining of bronchial epithelial cells (Figure 1A, Supplementary Figure 2, A and B). Similarly, ductal adenocarcinoma of the pancreas showed no or only weak ATGL protein expression while pancreatic duct epithelium was strongly positive for ATGL (Figure 1A, Supplementary Figure 2, A and B). Studying different stages of pancreatic intraepithelial neoplasia (PanIN), ATGL levels were already reduced in early preinvasive cancerous lesions, PanIN-1, and more so in later stage PanIN-3 (Figure 1A and 1B, Supplementary Figure 2, A and B). To test whether ATGL levels are also diminished in benign tumors, we studied smooth muscles tumors, leiomyoma. ATGL protein abundance in leiomyoma was high and similar to that observed in normal smooth muscle tissue (Figure 1A and 1C, Supplementary Figure 2, A and B). In contrast, ATGL staining was markedly decreased in leiomyosarcoma, the malignant counterpart of leiomyoma (Figure 1A and 1C, Supplementary Figure 2, A and B). These data indicate that ATGL expression is reduced or lost in non-small cell lung cancer and other types of malignancies suggesting that cancer cells, similar to fetal tissues, obtain the required energy for homeostasis through glucose and glutamine metabolism rather than from FA beta-oxidation. This is in line with the current concepts of cancer metabolism [5]. Consistent with this concept, we observed a negative correlation between ATGL and glucose transporter 1 (Glut1) expression during the progression of PanIN to invasive carcinoma (Supplementary Figure 3, A and B) indicating a switch from fatty acid to glucose utilization.

To reveal whether low ATGL expression in cancer was a more general phenomenon, we extracted data for *ATGL* mRNA expression levels from four datasets of The Cancer Genome Atlas (TCGA) database. In agreement with our results on ATGL protein levels, we found a highly significant decrease in *ATGL* mRNA levels in several forms of cancer compared to their matched normal tissue, including non-small cell lung cancer (Supplementary Figure 4). Upon analyzing a multi-cohort gene expression dataset, we found that low levels of *ATGL* mRNA are associated with poor survival in ovarian, breast, gastric and non-small cell lung cancer patients (Supplementary Figure 5). Importantly, considering all types of cancer, the *ATGL* gene is deleted in 24.7 % of tumor samples when evaluating copy number changes (*q-value* = 5.64×10^−72^). The *ATGL* gene is deleted in about 38% of lung cancer (*n* = 1016, *q-value* = 1.74×10^−13^), and in a significant percentage (*q-value* <.25) in 13 additional cancer types (Table 1).

**Table 1:** Analysis of *ATGL* gene copy number alteration across multiple cancer types collected in The Genome Cancer Atlas (TGCA)

Cancer subset	Amplifications		Deletions	
	Frequency	q-value	Frequency	q-value
**All cancers**	0.09	1.0	0.25	**5.64E-72**
**Epithelial cancers**	0.10	1.0	0.25	**3.18E-53**
**Ovarian serous cystadenocarcinoma**	0.10	1.0	0.59	**6.39E-31**
**Lung cancers**	0.12	1.0	0.38	**1.74E-13**
**Brain lower grade glioma**	0.06	1.0	0.21	**5.94E-13**
**Breast invasive adenocarcinoma**	0.13	1.0	0.28	**6.35E-12**
**Glial cancers**	0.04	1.0	0.22	**7.6E-12**
**Lung squamous cell carcinoma**	0.09	1.0	0.49	**5.32E-11**
**Uterine corpus endometrioid carcinoma**	0.07	1.0	0.19	**9.54E-8**
**Sarcoma**	0.09	1.0	0.42	**2.98E-6**
**Lung adenocarcinoma**	0.16	1.0	0.27	**0.00281**
**Glioblastoma multiforme**	0.04	1.0	0.23	**0.00765**
**Bladder urothelial carcinoma**	0.06	1.0	0.52	**0.0213**
**Esophageal carcinoma**	0.16	1.0	0.40	**0.0219**
**Stomach adenocarcinoma**	0.13	1.0	0.20	**0.0296**
**Pancreatic adenocarcinoma**	0.05	1.0	0.156	**0.103**
**Pheochromocytoma and Paraganglioma**	0.04	1.0	0.35	**0.126**
**Head and neck squamous cell carcinoma**	0.13	1.0	0.31	**0.244**
**Uterine carcinosarcoma**	0.11	1.0	0.46	0.412
**Adrenocortical carcinoma**	0.09	1.0	0.3	0.596
**Acute myeloid leukemia**	0.02	1.0	0.01	0.635
**Blood cancers**	0.06	1.0	0.01	0.884
**Cervical squamous cell carcinoma**	0.05	1.0	0.36	1.0
**Cutaneous melanoma**	0.12	1.0	0.31	1.0
**Melanomas**	0.12	1.0	0.26	1.0
**Liver hepatocellular carcinoma**	0.10	1.0	0.17	1.0
**Rectum adenocarcinoma**	0.2	1.0	0.15	1.0
**Kidney chromophobe**	0.23	1.0	0.14	1.0
**Colorectal cancers**	0.15	1.0	0.13	1.0
**Colon adenocarcinoma**	0.13	1.0	0.12	1.0
**Mesothelioma**	0.15	1.0	0.06	1.0
**Kidney renal papillary cell carcinoma**	0.04	1.0	0.06	1.0
**Kidney cancers**	0.07	1.0	0.05	1.0
**Prostate adenocarcinoma**	0.05	1.0	0.04	1.0
**Kidney renal clear cell carcinoma**	0.06	1.0	0.04	1.0
**Uveal melanoma**	0.13	1.0	0.03	1.0
**Diffuse large B-cell lymphoma**	0.21	1.0	0.02	1.0
**Thyroid carcinoma**	0.01	1.0	0.02	1.0

a Analysis version: 2014-11-03 stddata__2014_10_17; dataset of 10570 tumors

**Figure 2:**
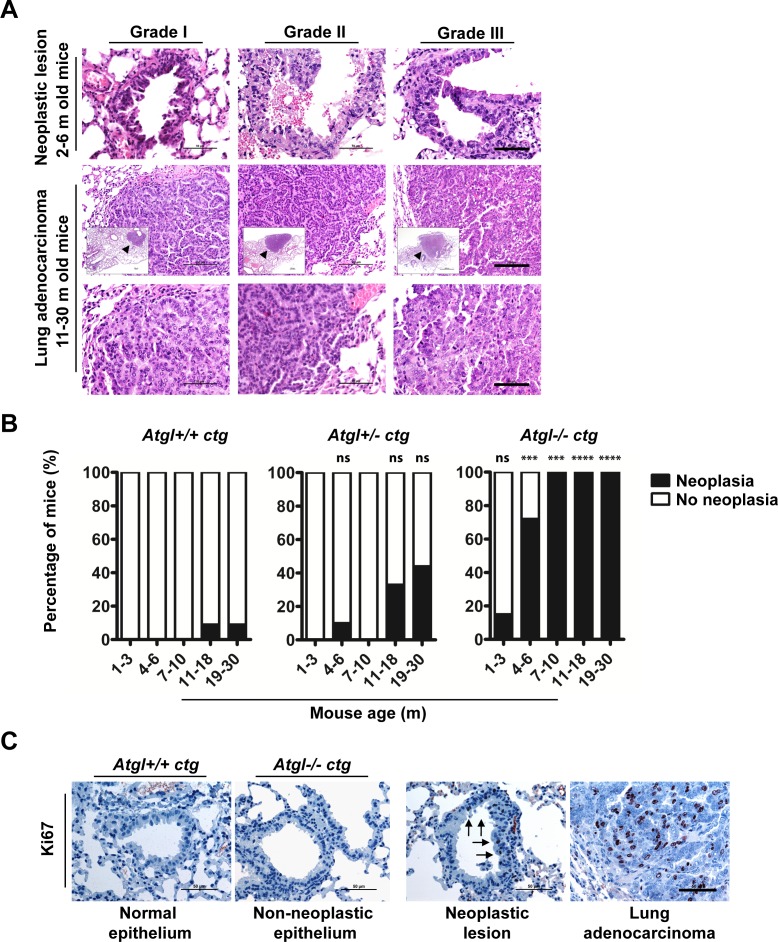
Loss of ATGL results in spontaneous pulmonary neoplasia **A.** Histopathological analysis of intraepithelial neoplastic lesions and invasive epithelial tumors (adenocarcinoma) in lungs of *Atgl+/−* ctg and *Atgl−/−* ctg mice. We did not detect lung adenocarcinoma in any of the mice from the three genotypes that were younger than 10 months. In mice older than 10 month, no lung adenocarcinoma were detected in *Atgl+/+* ctg mice (*n* = 22). Out of 20 *Atgl−/−* ctg mice, we detected adenocarcinoma in 5 mice (*P* value = .0182). We also detected adenocarcinoma in 4 out of 21 *Atgl+/−* ctg mice (*P* value = .0485). (Upper panels) show intraepithelial neoplastic lesions. Scale bar, 50 μm. (Middle panels) show invasive adenocarcinoma. (Inserts) show the entire tumor area (arrowheads). Scale bar, 100 μm. Representative tumor regions are shown at higher magnification in the (lower panels) demonstrating cellular atypia. Scale bar, 50 μm. **B.** Bar graphs show the percentage of mice in which one or more pulmonary neoplastic lesions were detected. *Atgl+/+* ctg mice, (*n* = 50); *Atgl+/−* ctg mice (*n* = 49); *Atgl−/−* ctg mice (*n* = 49). **C.** IHC analysis of *Atgl+/+* ctg, *Atgl+/−* ctg, or *Atgl−/−* ctg lung sections using antibodies against Ki67 (proliferation marker). Representative images show no Ki67 positive cells in non-neoplastic lung epithelium of *Atgl+/+* ctg and *Atgl−/−* ctg mice, whereas neoplastic lesion and adenocarcinoma show noticeable positive nuclei. Arrows highlight Ki67 positive nuclei and point to the part of a bronchiole with an intraepithelial neoplastic lesion. Scale bar, 50 μm.

To explore functional consequences of ATGL loss, we investigated whether ablation of this enzyme is associated with a higher risk of *de novo* tumor development. We aged ATGL deficient mice in which the cardiac muscle failure phenotype, associated with whole body knockout of *atgl*, has been rescued by cardiac myocyte specific transgenic (ctg) *atgl* expression [24, 25]. Evidence for increased tumor risk was provided by the occurrence of spontaneous lung tumors in *Atgl+/−* ctg and *Atgl−/−* ctg mice. Deficiency of both wild-type *Atgl* alleles (*Atgl−/−* ctg) provoked the development of multi-focal pulmonary neoplastic lesions early in life; within the first three months. *Atgl−/−* ctg mice older than 3 months developed significantly increased numbers of neoplastic lesions compared to *Atgl+/+* ctg mice (Figure 2A and 2B, Supplementary Table). Upon further aging (7-30 months), all *Atgl−/−* ctg mice developed pulmonary neoplastic lesions (Figure 2B, Supplementary Table). A gene dosage effect is apparent as *Atgl+/−* ctg mice developed substantially lower numbers of these lesions, which occurred later in life compared to those of *Atgl−/−* ctg mice (Figure 2B, Supplementary Table). In 25% of *Atgl−/−* ctg mice (5 out of 20), and in 19% of *Atgl+/−* ctg mice (4 out of 21) older than 10 months, these neoplastic lesions evolved into invasive adenocarcinoma (Figure 2A). In only 4% of *Atgl+/+* ctg mice (2 out of 50) we observed non-invasive neoplastic lesions consistent with the low rate of spontaneous lung tumor formation in the parental C57BL/6 mouse strain [26, 27] (Figure 2B, Supplementary Table). All neoplastic lesions showed cellular atypia and abundant proliferating cells (Figure 2A and 2C). Non neoplastic lung epithelium of mice form the three genotypes showed no positively stained nuclei for Ki67 (proliferation marker) (Figure 2C). In contrast, neoplasia and adenocarcinoma were characterized by Ki67 positive nuclei regardless of genotype (Figure 2C). The vast majority of the neoplastic lesions were located at the terminal bronchioles indicating that they might originate from epithelial cells localized at the bronchoalveolar duct junction. Epithelial cells at the bronchoalveolar duct junction showed lipid accumulation already in two months old *Atgl−/−* ctg mice —in line with defective lipolysis— whereas no Oil Red O positive cells were detected in *Atgl +/+* ctg controls (Supplementary Figure 6). Collectively, these data demonstrate that loss of ATGL increased the risk of lung tumor development in a time- and gene-dose dependent manner.

## DISCUSSION

Our study provides evidence that ATGL, the rate limiting enzyme in the catabolic arm of TG/FA cycling, functionally acts as a tumor suppressor. ATGL protein and mRNA levels were low to undetectable in non-small cell lung cancer and several other types of human cancer. Remarkably, ATGL was already reduced or lost in non-invasive pre-cancerous lesions of the pancreatic duct epithelium (PanIN), indicating that a reduction of cellular ATGL protein levels occurs early during pancreatic carcinogenesis. Moreover, ATGL protein levels in benign smooth muscle tumors, leiomyoma, were similar to the corresponding normal tissue. In contrast, the malignant counterpart, leiomyosarcoma, had significantly lower levels. These results suggest that ATGL might be a potent immunohistochemical marker for diagnostic surgical pathology ─the potential usefulness of ATGL expression in combination with other markers has yet to be evaluated.

The *ATGL* gene is located within the chromosomal band 11p15.5. Since alterations in this region have been implicated in a broad variety of childhood and adult malignancies, including lung cancer, it is considered to be a critical tumor-suppressor gene region [28–33]. Importantly, copy number variation data deposited in TCGA indicate a deletion of the ATGL gene in 24.7% of samples when all forms of cancer are taken into account. To be considered as a “classical” tumor suppressor gene, the number of point mutations reported is, however, too small (COSMIC database). Nevertheless, our experimental data in an animal model system suggest a tumor suppressive function for ATGL because loss of the *Atgl* gene induces the development of spontaneous pulmonary neoplasia ─which can progress to adenocarcinoma.

Consistent with our findings, a similar association has recently been reported for the ATGL coactivator protein ABHD5 in colorectal cancer [20], underlining the importance of the regulation of lipolysis in malignant tumors. In addition, MGL, the enzyme that catalyzes the final step in the hydrolysis of triglycerides, has been shown to regulate a fatty acid network that promotes cancer pathogenesis [19]. Together, these studies and our data illustrate the importance of the lipolytic pathway in cancer.

Our work does not provide answers for two questions. First, what is/are the molecular mechanism(s) involved in the observed ATGL reduction in human cancers (Figure 1) Even though the ATGL gene is frequently deleted in several cancers (see Table 1), other factors and conditions may account for the reduction in ATGL protein expression, that we observed in various cancers (Figure 2). In fact, ATGL abundance is regulated by multiple players including members of the insulin signaling network, which are reported to be frequently altered in cancer cells [34–36]. Second, what are the mechanisms leading to the development of lung neoplastic lesions in ATGL knockout mice? Additional studies are required to address these questions and we are currently engaged in this endeavor.

Because ATGL is reduced or lost in human malignant epithelial and mesenchymal tumors and ATGL deficiency induces pulmonary neoplasia in an animal model, we propose that ATGL qualifies as a tumor suppressor. The well-defined function as a lipase suggests that ATGL might act as a metabolic tumor suppressor. Lipogenic pathways and *de novo* lipogenesis have already been considered as potential pharmaceutical targets in human malignancies. This work suggests a possible role of the lipolytic cascade in cancer prevention and might open up additional diagnostic and therapeutic avenues.

## MATERIALS AND METHODS

### Immunohistochemistry (IHC) and haematoxylin/eosin (H&E) staining

The analysis of normal and tumor tissues retrieved from the Biobank of the Medical University of Graz was approved by the ethics committee of the Medical University of Graz (Number: 21-244 ex 09/10; 23-279 ex 10/11). Formalin-fixed, paraffin-embedded tissue samples were sectioned, and stained with H&E according to standard histopathological techniques [37]. For ATGL IHC, sections were incubated with anti-human ATGL antibody (Cat#3814-100, Biovision, Milpitas, CA, USA, 1:50 dilution). Antibody binding was visualized using aminoethyl carbazole (AEC) substrate chromogen (cat#3464, Dako, Glostrup, Denmark). For Glut1 IHC, sections were incubated with anti-human Glut1 antibody (Cat#MS-10637-R7, Thermo scientific, Fermont, CA, USA, 1:400 dilution). Antibody binding was visualized using EnVision+ System-HRP (DAB) (code K4006, Dako, CA, USA). For ATGL/Glut1 IHC double staining, sections were incubated with anti-human Glut1 antibody and with anti-human ATGL antibody. Antibody binding was visualized using MACH 2 Double Stain 2 (cat# MRCT525 G, H, L, Biocare Medical, concord, CA, USA) according to the manufacturer's instructions. For IHC in mouse lung tissues we used mouse specific Ki67 antibody (cat#NCL-Ki67p, Novocastra, Newcastle, UK, 1:50 dilution). Antibody binding was visualized using AEC (cat#3464, Dako, Glostrup, Denmark). Tissue samples were counter stained with haematoxylin according to standard methods [38].

### Evaluation of ATGL Immunohistochemistry

ATGL IHC intensity and percentage of stained cells from normal and cancer tissues were analyzed using the histoscore (H-Score) method [39]. The intensity (I) of cytoplasmic staining was classified into four grades (0, 1, 2, and 3). The percentage (P) of cells within each category was estimated by pathologists. Normal and tumor tissue were evaluated and the H-score for each sample was calculated according to the formula: *H-score* = [(I_(0)_xP_(0)_)+(I_(1)_xP_(1)_)+(I_(2)_xP_(2)_)+(I_(3)_xP_(3)_)].

### Data mining

Gene expression analysis of *ATGL* mRNA datasets of matched tumor and normal tissue (downloaded from The Cancer Genome Atlas (TCGA)) was performed with the R (version 3.02) package DESeq2 version 1.6.3. The *P* values were corrected for multiple testing using the Benjamini-Hochberg False Discovery Rate (Adjusted *P* values). Overall survival analyses in breast, ovarian, gastric and non-small cell lung cancer patients were performed as described [40]. The online available software tool combines Affymetrix gene expression data from multiple annotated cancer studies into a single database which can be then queried for associations of patient outcome with the expression of individual genes. Gene copy number alteration data from TCGA projects were generated at the Broad institute (Cambridge, MA, USA) and were analyzed as described [41].

### Animal care

*Atgl*+/+ ctg, *Atgl+/−* ctg and *Atgl−/−* ctg mice were generated in the laboratory of R.Z [24, 25]. All animals were housed in filter-top plastic cages and maintained on a regular light-dark cycle (12 h light, 12 h dark) and kept on a standard laboratory chow diet (4.5% w/w fat). During experiments, food intake as well as body weight was controlled for animal well-fare. All animal studies were performed in accordance with the guidelines and provisions of the Commission for Animals Experiments of the Austrian Ministry of Science (Animal license numbers: BMWF-66.010/0140-II/3b/2012; BMWF-66.010/0025-II/3b/2012; BMWF-66.010/0013-II/3b/2010).

### Lung tumor assessment

*Atgl+/+* ctg, *Atgl+/−* ctg, and *Atgl−/−* ctg mice were maintained as described above. At defined age, mice were sacrificed by isoflurane inhalation. The thoracic cavity was opened, and the trachea was exposed. Lungs were resected, inflated and fixed with 4% formaldehyde. After paraffin embedding, sections were used for H&E staining and IHC using standard techniques. At least five sections were stained for H&E from each block and evaluated for the presence of neoplastic lesions by a pathologist. For Oil Red O staining, lung tissues were collected and stored in liquid nitrogen. Frozen sections were stained with Oil Red O according to standard protocols.

### Statistical analysis

Data are presented as means and all error bars represent standard deviation of the mean (SD). Statistical analysis was performed using Graphpad Prism version 5 and *P* values were obtained using an unpaired t-test, unless otherwise stated. Fisher's exact test was used in the analysis of lung neoplasia and adenocarcinoma. *P* values < .05 were considered statistically significant. All statistical analyses were two-sided. For somatic copy number analysis *q*-values are the false discovery.

## SUPPLEMENTARY MATERIALS FIGURES AND TABLES


