# Supplementary material for: Loss of adipose triglyceride lipase is associated with human cancer and induces mouse pulmonary neoplasia
Source: Oncotarget. 2016 May 17;7(23):33832–40. doi: 10.18632/oncotarget.9418 (PMC5085122; doi:10.18632/oncotarget.9418)
Supplement: Supplementary file 1 [file oncotarget-07-33832-s001.pdf]

Loss of adipose triglyceride lipase is associated with human cancer and induces mouse pulmonary neoplasia

Supplementary Material

This file includes:

- **Supplementary Figures 1- 6**
- **Supplementary Table**

A

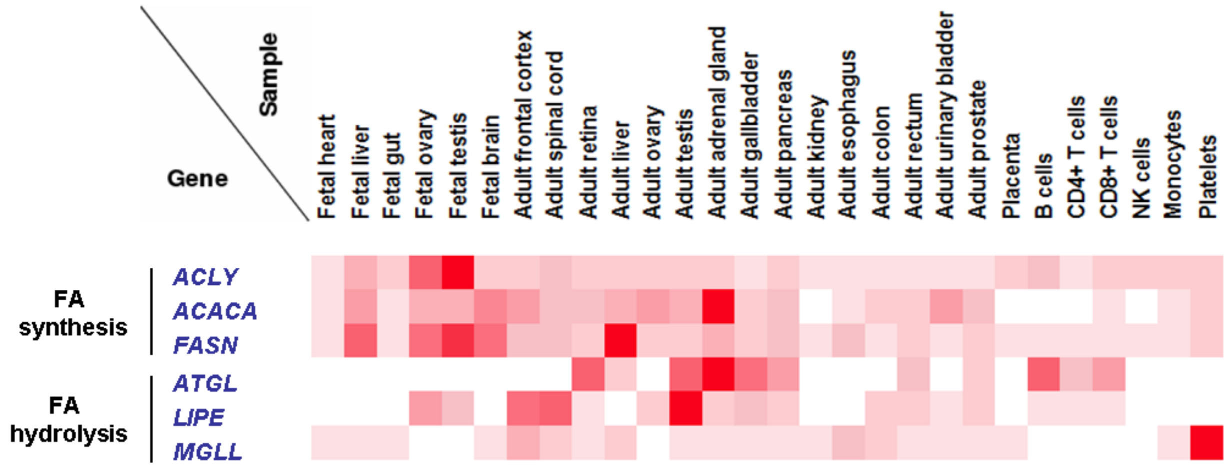

B

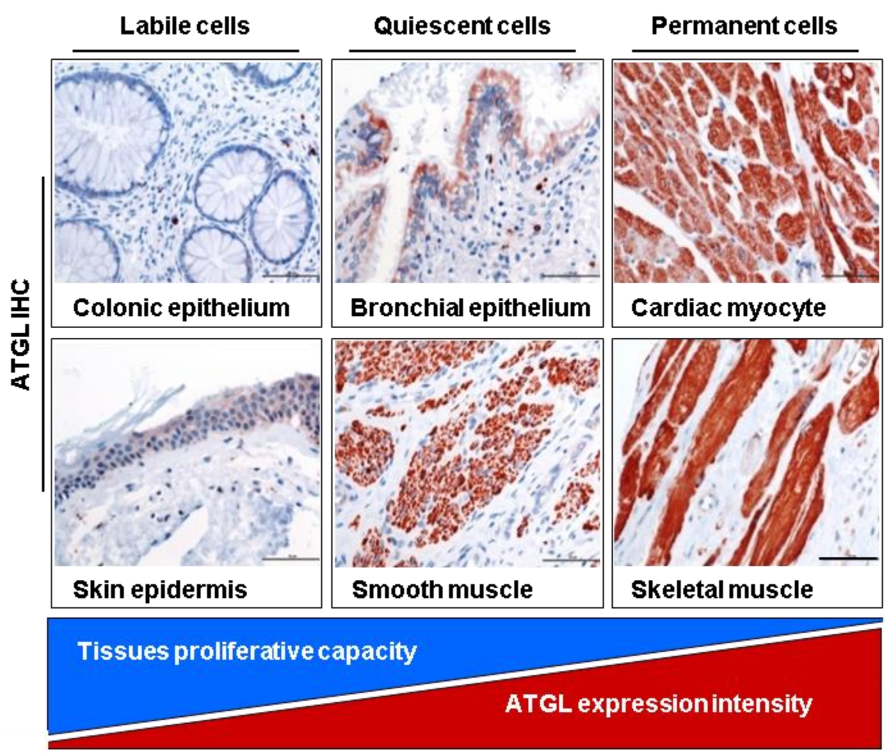

Supplementary Figure 1. ATGL protein expression. A. Heat map of protein abundance in fetal and adult tissues. FA, fatty acids; *ACLY*, ATP citrate lyase; *ACACA*, acetyl-CoA carboxylase alpha; *FASN*, fatty acid synthase; *ATGL*, adipose triglyceride lipase (the full name is patatin-like phospholipase domain containing 2, *PNPLA2*); *LIPE*, lipase hormone-sensitive (also known as *HSL*); *MGLL*, monoglyceride lipase (also known as *MGL*). **B.** ATGL protein expression across normal tissues. Scale bar, 50μm.

A

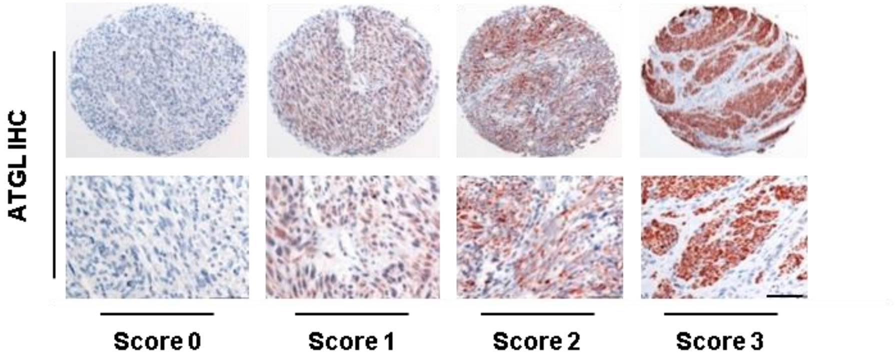

B

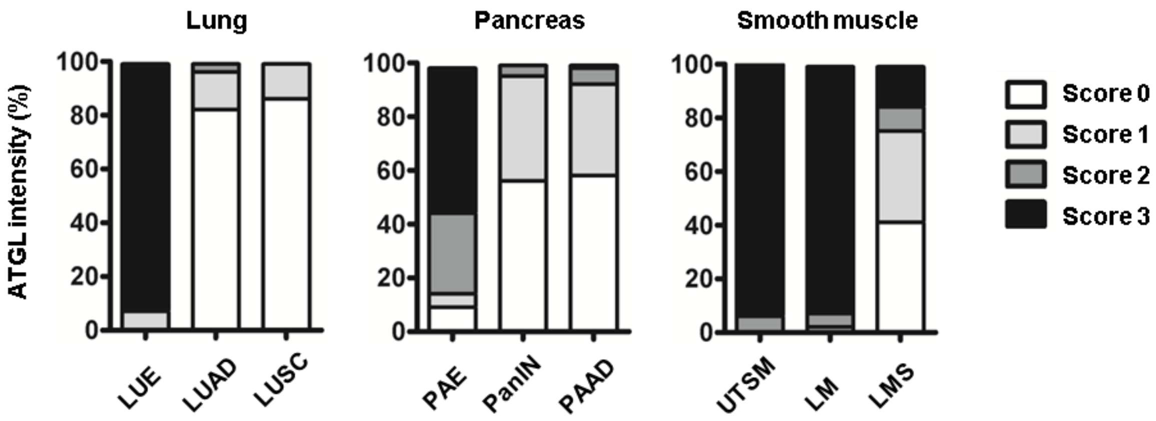

Supplementary Figure 2. ATGL protein is frequently suppressed in human cancer. **A**. Images illustrating ATGL IHC staining intensity score. Regions from the upper panels are shown in higher magnification in the lower panels. Scale bar, 50µm. **B**. Bar graphs show percentages of ATGL IHC staining intensity in neoplasia of lung epithelium, pancreatic epithelium or smooth muscle (**see Fig. 1**). LUE, normal lung epithelium; LUAD, lung adenocarcinoma; LUSC, lung squamous cell carcinoma; PAE, normal pancreatic epithelium; PanIN, pancreatic intraepithelial neoplasia; PAAD, pancreatic adenocarcinoma; UTSM, normal uterus smooth muscle; LM, benign smooth muscle tumor (leiomyoma); LMS, malignant smooth muscle tumor (leiomyosarcoma).

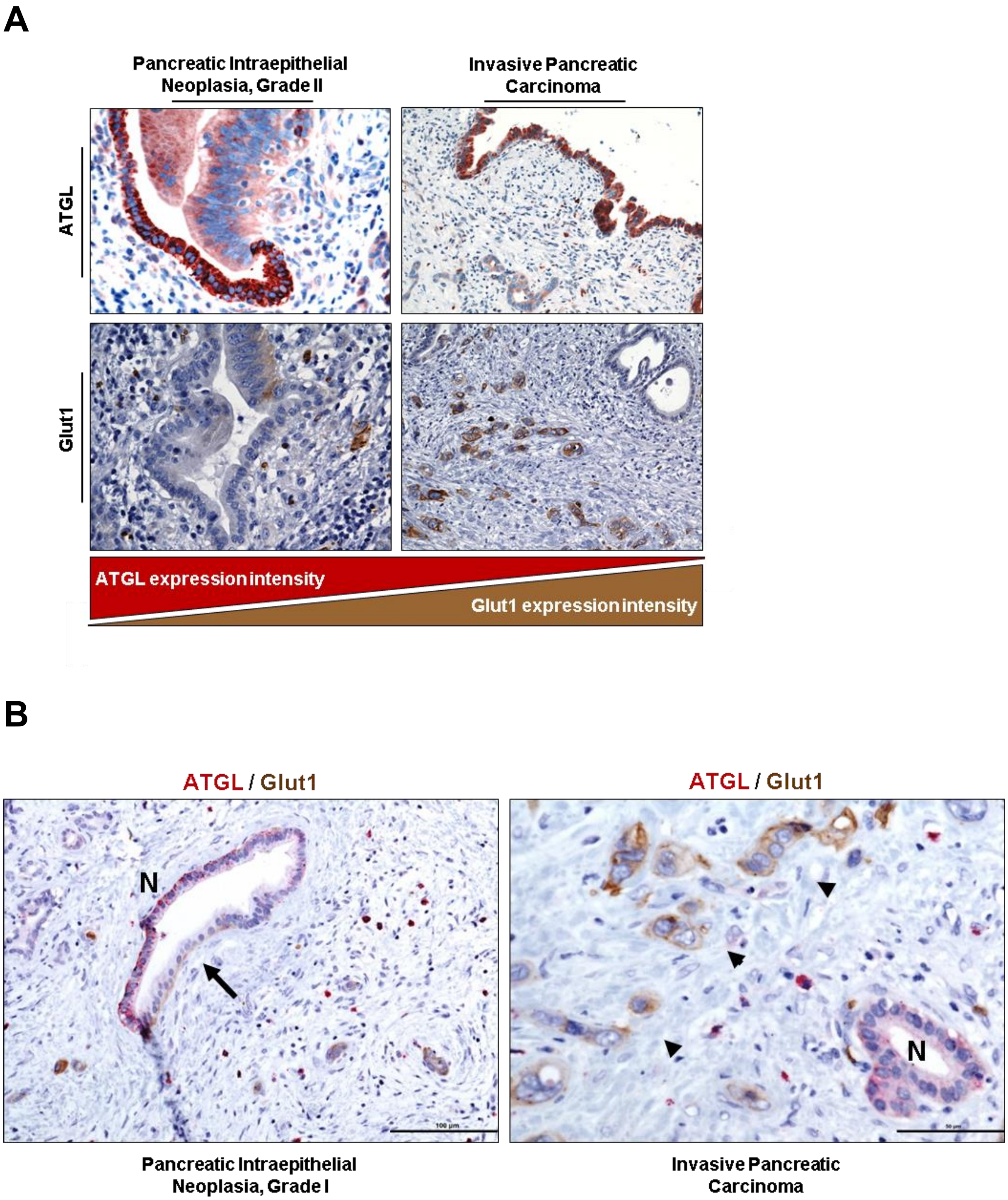

Supplementary Figure 3. Inverse correlation between ATGL and glucose transporter 1 (Glut1). A. Immunohistochemical (IHC) staining for ATGL (upper panels), or Glut1 (lower panels)**. B**. Double IHC staining for ATGL (signal in red) and Glut1 (signal in brown). N, normal epithelium; arrow points to a neoplastic lesion shows detectable Glut1 signal at the basal domain; arrowheads point to invasive tumor cells. Scale bars, 100μm (left panel) and 50μm (right panel).

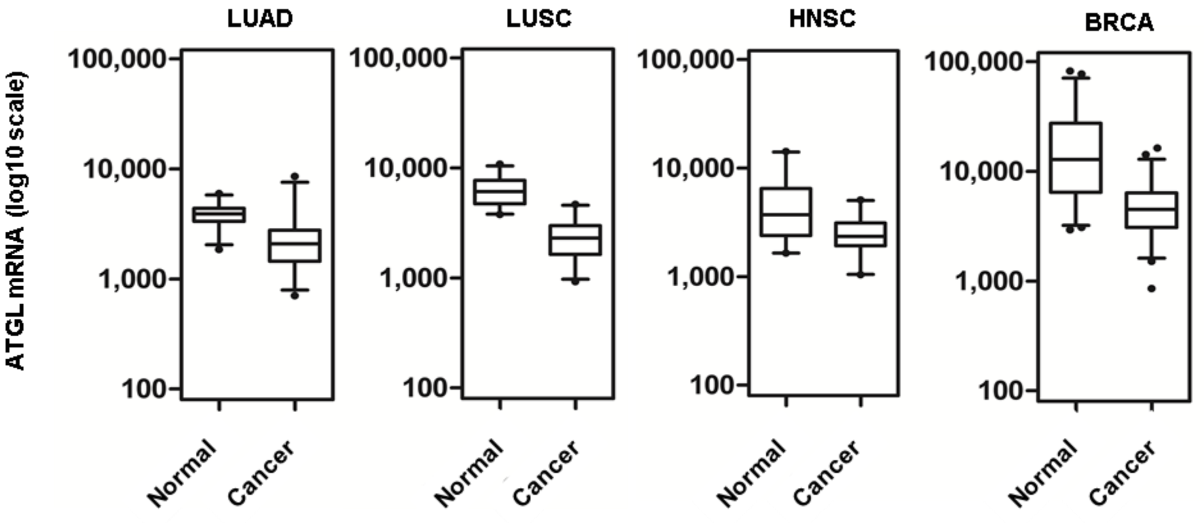

Supplementary Figure 4. *ATGL* mRNA expression levels in samples of four types of cancer patients. LUAD, lung adenocarcinoma; LUSC, lung squamous cell carcinoma; HNSC, head and neck squamous cell carcinoma; BRCA, breast ductal carcinoma. (LUAD: *n* = 57, Log2fold change = -0.85, Adjusted *P* value = $1.47X10^{-17}$; LUSC: *n* = 50, Log2fold change = -1,41, Adjusted *P* value = $6.23x10^{-50}$; HNSC: *n* = 40, Log2fold change = -0.72, Adjusted *P* value = $1.57X10^{-7}$; BRCA: *n* = 108, Log2fold change = -1,61, Adjusted *P* value = $6.17x10^{-33}$.)

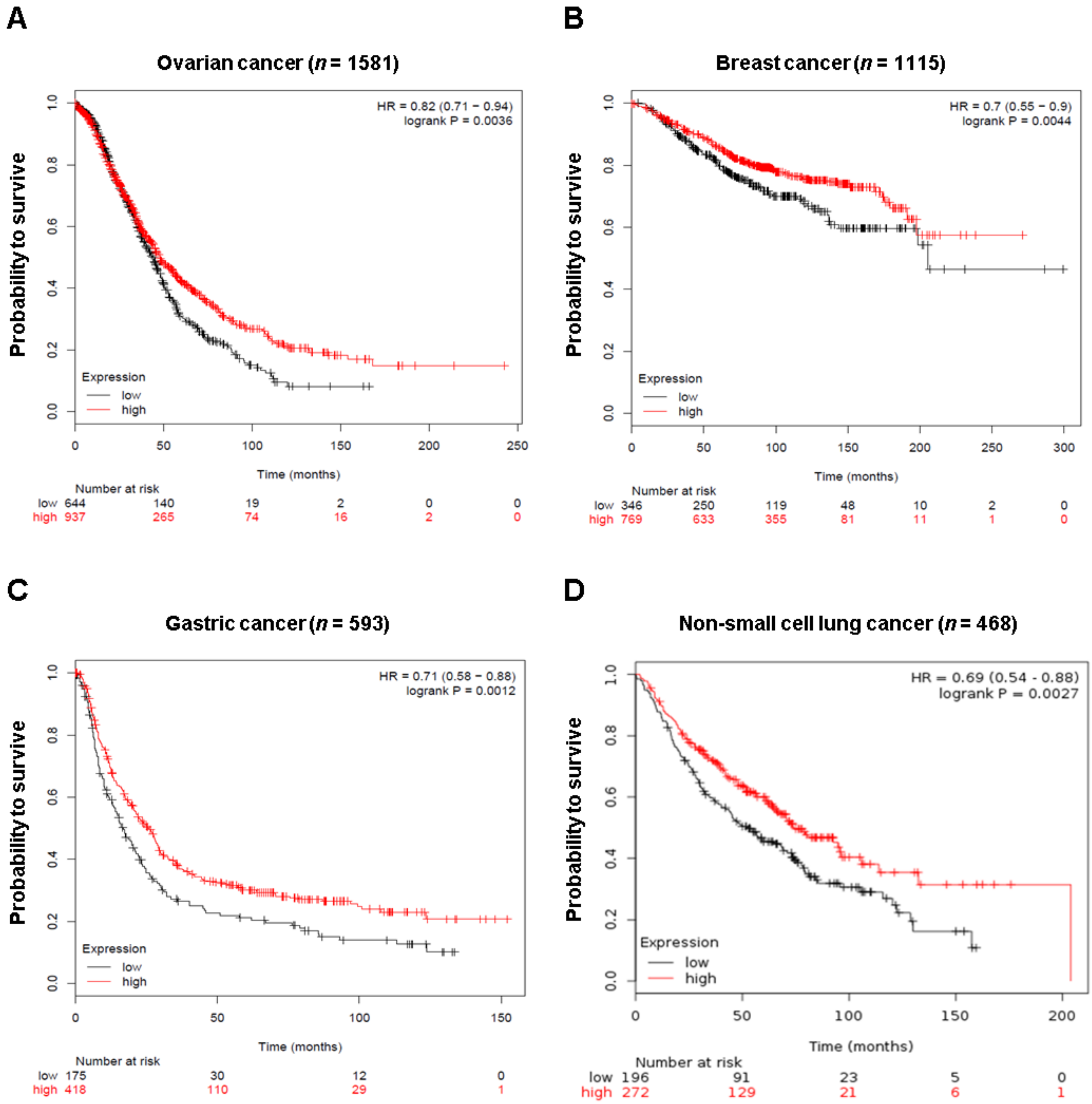

Supplementary Figure 5. *ATGL* mRNA expression level is a prognostic factor in multiple cohorts of different types of cancer. Overall survival of 1581 ovarian cancer patients (**A**), 1115 breast cancer patients (**B**), 593 gastric cancer patients (**C**), 486 non-small cell lung cancer patients (CAARRAY cohort) (**D**) after dichotomization according to the ATGL expression levels. HR: hazard ratio.

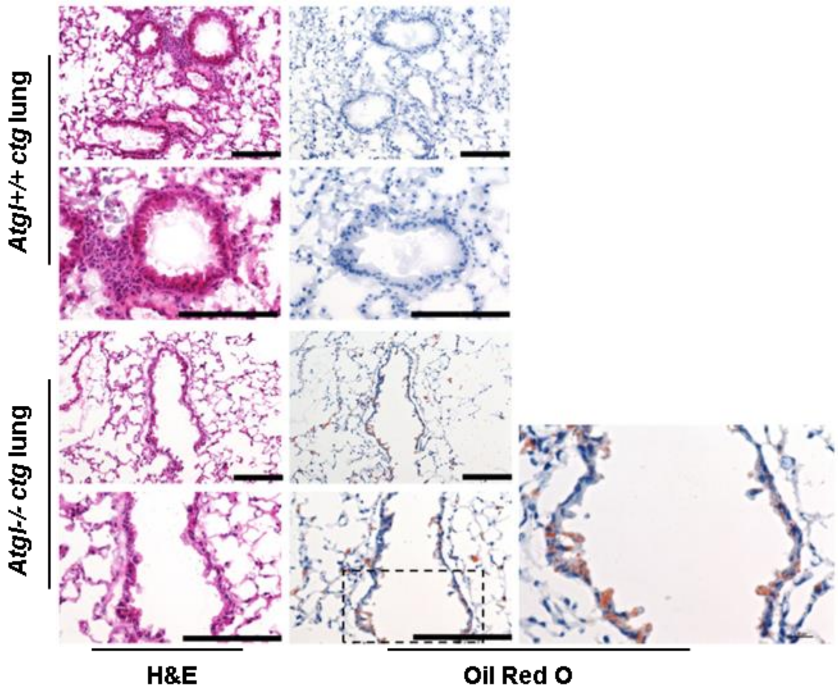

Supplementary Figure 6. Accumulation of neutral lipids in ATGL deficient bronchial epithelial cells. Representative images show H&E and Oil Red O staining of lung sections. Bronchial epithelium of *Atgl-/-* ctg mice stains positive for Oil Red O whereas bronchial epithelium of *Atgl+/+* ctg mice is negative. Regions from the upper panels are shown in higher magnification in the lower panels. Scale bar, 100µm.

Supplementary Table: Number of animals with neoplastic lesions detected in lungs of *Atgl+/+* ctg, *Atgl +/-* ctg and *Atgl -/-* ctg mice

Age group (months)	Number of animals with pulmonary neoplasia Neoplasia/Total(Statistical significance)		
	Atgl **+/+ ctg**	***Atgl*** **+/- ctg**	***Atgl*** **-/- ctg**
**1-3**	0/8	0/13 (ns)	2/13 (ns)
**4-6**	0/10	1/10 (ns)	8/11(***)
**7-10**	0/10	0/5 (ns)	5/5 (***)
**11-18**	1/11	4/12 (ns)	12/12 (****)
**19-30**	1/11	4/9 (ns)	8/8 (****)

[a] Symbol meanings: ^{ns} *P* > .05; ****P* ≤ .001; *****P* ≤ .0001

[b] Frequency of neoplasia in *Atgl+/-* ctg *or Atgl-/-* ctg mice is compared to frequency of neoplasia in *Atgl+/+* ctg mice
